# An Unusual Presentation of a Cardiac Foreign Body in a Pediatric Patient

**DOI:** 10.7759/cureus.4829

**Published:** 2019-06-04

**Authors:** Mohammed Al-Musawi, David Rubay, Levonti Ohanisian, Angel Sidley, Ali N Abed

**Affiliations:** 1 Surgery, Anschutz Medical Campus, University of Colorado, Aurora, USA; 2 Surgery, Charles E. Schmidt College of Medicine, Florida Atlantic University, Boca Raton, USA; 3 Orthopaedic Surgery, Charles E. Schmidt College of Medicine, Florida Atlantic University, Boca Raton, USA; 4 Pediatrics, Charles E. Schmidt College of Medicine, Florida Atlantic University, Boca Raton, USA; 5 Cardiac Surgery, Iraqi Center for Heart Diseases/Medical City Teaching Complex, Baghdad, IRQ

**Keywords:** cardiac foreign body, foreign body

## Abstract

Cardiac foreign bodies (FBs) are rare. Their etiology can be attributed to penetrating injuries although they are also often found incidentally. The approach for removal of these FBs is variable and patient dependent. Although there is debate regarding indications for removal, there is a general consensus that symptomatic FBs presenting acutely, as well as asymptomatic FBs posing a greater risk of complication to the patient, should be removed. We present the case of a 14-year-old patient with a cardiac FB and a step-wise approach for removal.

## Introduction

Cardiac foreign bodies (FB) are rare with only a limited number of cases reported in the literature [1]. They may be due to penetrating injuries [2], diagnosed incidentally, or found on workup relating to complications of the FB [1,3-4]. In the case of the latter, patients are often referred to cardiothoracic surgery from other services including, but not limited to, cardiology and gastroenterology [4-6]. There is a consensus that symptomatic FBs presenting acutely and asymptomatic FBs that pose a heightened future risk to the patient should be removed [2,7]. However, debate still exists regarding the removal of FBs where the diagnosis of such is delayed and not associated with significant future risk [2].

## Case presentation

**Figure 1:**
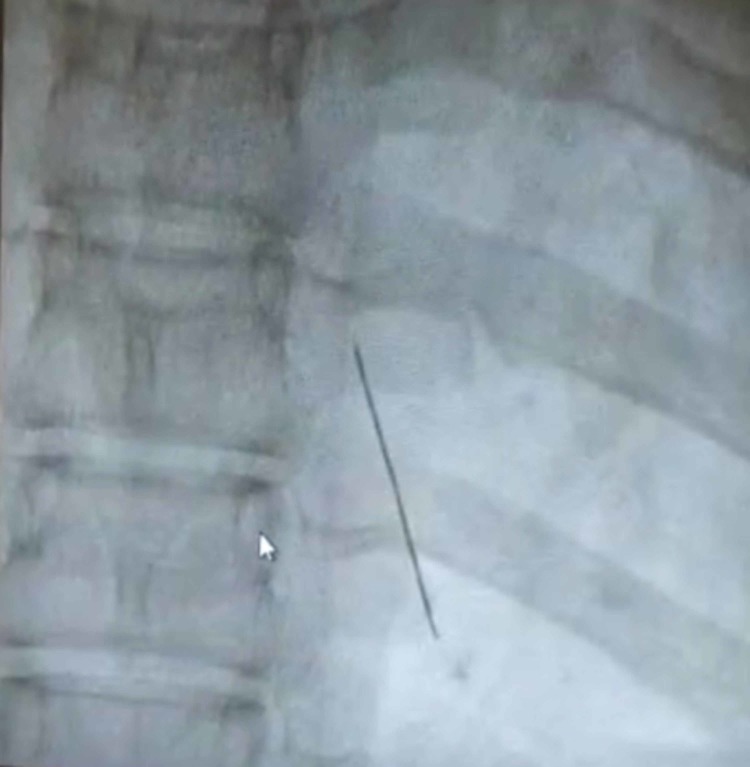
Foreign body (anterior view)

**Figure 2:**
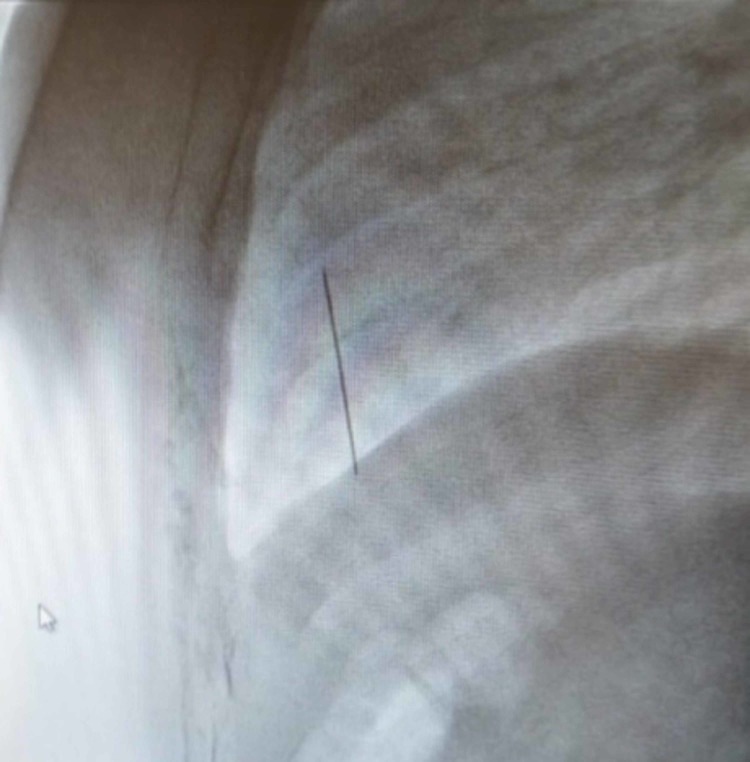
Foreign body (lateral view)​

The patient is a 14-year-old female with no significant past medical history or known trauma who presented with fever and a dry cough. She was initially treated for respiratory tract allergic bronchitis for 3-4 months. Due to a lack of improvement and the recent development of shortness of breath at rest, a chest X-ray (CXR) was ordered demonstrating a globular shadow of pericardial effusion. The patient was then referred to a cardiologist who performed a subsequent echocardiogram to assess the etiology of the pericardial effusion. The echocardiogram demonstrated moderate-to-severe pericardial effusion and tamponade. The patient was immediately taken to the catheterization suite for percutaneous drainage of the effusion under fluoroscopy. At this time, the cardiologist noted that there was an intracardiac metallic foreign body located behind the sternum in the wall of the right ventricle, moving with each heartbeat (Figures 1-2). 

**Figure 3:**
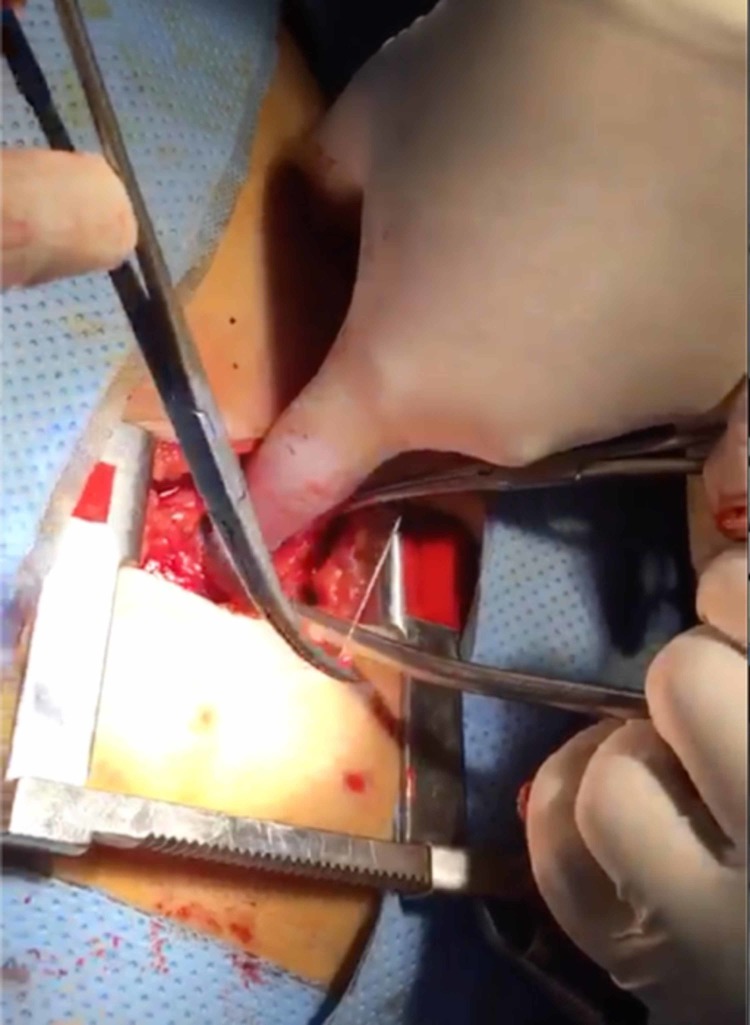
Foreign body removal

**Figure 4:**
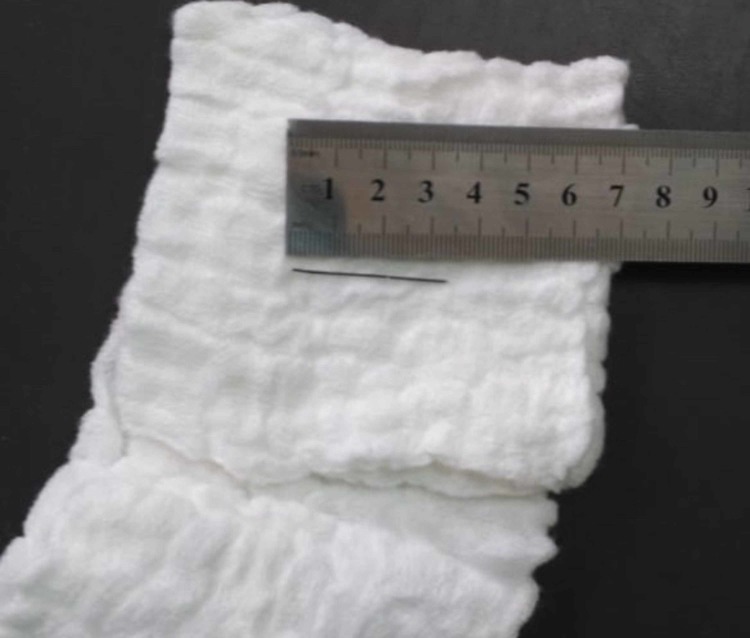
The foreign body scale

Cardiac surgery was consulted during the procedure and the decision was made to drain the effusion surgically via a subxiphoid approach under fluoroscopy and to assess whether the foreign body could be retrieved in the same manner. Preparations were made to convert to conventional median sternotomy under cardiopulmonary bypass if needed. After incising the pericardium from the subxiphoid incision and draining the hemorrhagic effusion, the surface of the heart was palpated under fluoroscopic guidance. We were able to visualize the tip of the foreign body that was emerging from the anterior wall of the right ventricle. The sternum was retracted superiorly to improve visualization and the surgeon used his index finger to press on the area around the tip of the foreign body in an effort to force it to protrude, so that it could then be grasped by forceps. Ultimately the FB was removed from the right ventricular wall using artery forceps, carefully pulled out in the same axis of its insertion to avoid causing any further damage. The FB was found to be an intact domestic sewing needle (Figures 3-4). Subsequent fluoroscopy revealed no additional foreign bodies inside or around the heart.

A sample of the pericardial effusion fluid was sent for gram stain, culture, and sensitivity to tailor the post-operative antibiotics accordingly. The pericardial space was washed out with normal saline, a pericardial drain was inserted, and the wound was closed around the drain. The patient was taken to the cardiothoracic intensive care unit (CTICU) and started on intravenous antibiotics. The following day, CXR and echocardiography were performed ensuring that there was no collection present and that no damage was caused to the tricuspid valve or the interventricular septum intraoperatively. The patient was determined to be stable and transferred to the surgical floor for further recovery and monitoring. After five days, she was discharged home with complete resolution of her symptoms.

## Discussion

Cardiac FBs are uncommon [1] with no standard guidelines for management [5]. However, there is a general consensus that symptomatic cardiac FBs should be removed [2]. The patient in our case was symptomatic and began demonstrating complications due to the FB which necessitated immediate intervention. The surgical approach for removal is individualized in the management of cardiac FB and is influenced mostly by the location of the FB in the heart and by the associated injuries caused by the FB [8]. Different surgical approaches are used depending on the clinical scenario including median sternotomy, and thoracotomy with and without cardiopulmonary bypass [1-3]. We recommend a step-wise approach, as evidenced in the management of our patient, beginning with a small incision while being prepared to convert to a more invasive approach if necessary. Since there is no standard surgical incision which would serve the purpose of retrieving a FB from the heart, we reason that it would be better to begin with a smaller incision to assess the feasibility of completing the procedure. Care should be taken to ensure that no piece of the FB is left behind and that there are no other FBs present [9]. What remains uncertain in our case is how the FB was introduced into the patient’s heart. There is literature that has associated similar scenarios with psychiatric illness [10]; however, our patient had no indication of a psychiatric condition.

## Conclusions

Cardiac FBs are uncommonly encountered. They may present with characteristic signs and symptoms, or they may be discovered incidentally. Management of cardiac FBs is individualized based on patient symptoms, location of the FB, and associated cardiac injuries.
